# Clinicopathological and molecular analysis of microsatellite instability in prostate cancer: a multi-institutional study in China

**DOI:** 10.3389/fonc.2023.1277233

**Published:** 2023-10-13

**Authors:** Huizhi Zhang, Xiaoqun Yang, Jialing Xie, Xiao Cheng, Jiayi Chen, Miaomiao Shen, Wenyi Ding, Suying Wang, Zhe Zhang, Chaofu Wang, Ming Zhao

**Affiliations:** ^1^ Department of Pathology, Ningbo Clinical Pathology Diagnosis Center, Ningbo, China; ^2^ Department of Pathology, Ruijin Hospital, Shanghai Jiaotong University School of Medicine, Shanghai, China; ^3^ Department of Pathology, Zhejiang Provincial People’s Hospital, People’s Hospital of Hangzhou Medical College, Hangzhou, China

**Keywords:** DNA repair, DNA mismatch repair, high throughput nucleotide sequencing, microsatellite instability, prostatic neoplasms

## Abstract

**Background:**

Microsatellite instability (MSI), or mismatch repair-deficiency (dMMR), is rare in prostate cancers (PCas). The histological and molecular features of PCas with MSI/dMMR are incompletely described. Thus, we sought to identify the characteristics of PCas with MSI/dMMR.

**Methods and results:**

We analyzed 1,141 primary treatment-naive PCas by MMR-related protein immunohistochemistry (MLH1, PMS2, MSH2, and MSH6). We identified eight cases exhibiting MSI/dMMR (0.7%, 8/1141). Of these, six tumors had both MSH2 and MSH6 protein loss, one had both MLH1 and PMS2 protein loss, and one had only MSH6 loss. Histologically, MSI/dMMR-PCas frequently demonstrated high histological grade (Grade Group 4 or 5), ductal/intraductal histology (6/8 cases), pleomorphic giant-cell features (4/8 cases), and conspicuous tumor lymphocytic infiltration (8/8 cases). Polymerase chain reaction-based analysis of seven MSI/dMMR tumors revealed two MSI-H tumors with loss of both MSH2 and MSH6 proteins. Subsequently, the seven cases underwent next-generation sequencing (NGS) analysis with a highly validated targeted panel; four were MSI. All cases had a high tumor mutation burden (median: 45.3 mutations/Mb). Overall, the MSI/dMMR-PCas showed a high frequency of DNA damage-repair pathway gene changes, including five with pathogenic somatic or germline MMR gene mutations. Activating mutations in the MAPK pathway, PI3K pathway, and WNT/β-catenin pathway were common. *TMPRSS2::ERG* rearrangement was identified in one case (1/7, 14.3%).

**Conclusions:**

Several pathological features are associated with MSI/dMMR in PCas. Identification of these features may help to select patients for genetic screening. As MSI/dMMR-PCas are enriched for actionable mutations, patients should be offered NGS to guide standard-of-care treatment.

## Introduction

Mismatch repair (MMR) pathways play critical roles in maintaining genomic fidelity during DNA replication. MMR deficiency (dMMR) is triggered by germline, somatic, and epigenetic changes in MMR genes (most commonly *MLH1*, *PMS2*, *MSH2*, and *MSH6*), which inactivate these genes, causing loss of MMR-related protein expression, as noted by immunohistochemistry (IHC), and microsatellite instability (MSI) development (1–3). Most cancers with MSI occur sporadically, but about 16% result from inherited mutations (Lynch Syndrome) (4). Recent studies using large-scale genome analyses have found that MSI occurs in virtually all cancer types at some frequency (5, 6). MSI has been used as a surrogate marker for dMMR. Knowing the MSI status, or dMMR, can help to identify patients with susceptibility to immune checkpoint inhibitors (ICIs), which is associated with a greater and more durable treatment response (6, 7). Accordingly, in 2017, the US Food and Drug Administration approved the PD-1 inhibitor Pembrolizumab for the treatment of all advanced cancers with MSI-high (H) or dMMR, irrespective of the site of tumor origin (8). Therefore, MSI testing in all cancer types has become increasingly important. Recently, MSI-H/dMMR prostate cancer (PCa) patients have also been reported to benefit from treatment with ICIs inhibiting PD-1 (9, 10).

MSI/dMMR is a common and well-defined feature of colorectal and endometrial adenocarcinoma (11–13). Reports on MSI in PCa have come to different conclusions. Using different detection methods, several studies have shown that MSI/dMMR was observed in approximately 1.2%–12% of PCa patients (6, 9, 14, 15). The histological and molecular features of dMMR in PCa remain incompletely described. With advances in molecular sequencing, several investigators have correlated dMMR with an aggressive phenotype and late-stage PCas and have noted that patients with certain features may have MSI/dMMR, including unusual metastatic sites (such as the lungs), high-grade Gleason scores, or variant histologies, such as ductal/intraductal PCa (10, 14, 16–19). Currently, the clinicopathological significance of MSI/dMMR in PCa is not fully understood and the underlying mechanisms of MSI/dMMR in PCa deserve further investigation.

Here, we investigated the prevalence, and the clinicopathological and molecular characteristics of MSI/dMMR-PCa in the Chinese population, with particular attention to potential clinicopathological features that may alert clinicians to consider MSI or genetic investigations.

## Material and methods

### Patients and samples

We retrospectively reviewed cases of PCa evaluated by IHC to assess MMR-related proteins loss, from January 1, 2019, to January 31, 2021, at Ningbo Clinical Pathology Diagnosis Center, Shanghai Ruijin Hospital, and Zhejiang Provincial People’s Hospital. A total of 1141 primary treatment-naive PCas were identified. Overall, 665 PCa cases (including Grade group (GG) 1: 27, GG2: 155, GG3: 71, GG4: 247, GG5: 165) were identified at the Ningbo Clinical Pathology Diagnosis Center, 400 PCa cases were identified at the Department of Pathology of Shanghai Ruijin Hospital (including GG1: 47, GG2: 171, GG3: 100, GG4: 23, GG5: 59), and 76 PCa cases were identified at the Department of Pathology of Zhejiang Provincial People’s Hospital (including GG1: 14, GG2: 27, GG3: 19, GG4: 9, GG5: 7).

Eight PCa patients with loss of MMR-related proteins were identified (0.7%, 8/1,141). All cases involved primary tumors and were sampled on transurethral resection of the prostate (TURP) (n = 1), needle biopsy (n = 1), or radical prostatectomy (n = 6). Hematoxylin and eosin-stained sections of these eight cases were reviewed independently by three experienced urological pathologists (H.Z, X.Y, and M.Z) to assess the histological type, Gleason score, the presence of lymphovascular invasion (LVI) and perineural invasion (PNI), and the presence of ductal/intraductal histology, a pleomorphic giant-cell component, tumor infiltrating lymphocytes (TILs), and locoregional lymph node metastases. TILs were considered to be “significant” when 10 TILs were identified per high-power field.

For the eight MSI/dMMR-PCa patients, we collected the clinical history, surgical procedure, and clinicopathological data by review of the medical records and pathology reports. Follow-up data were collected by telephonic interview. The study was approved by our institutional review board.

### Mismatch repair protein immunohistochemistry and interpretation

MMR-related protein IHC was performed using a BenchMark autostaining system (Roche, Basel, Switzerland), with appropriate controls. We used a mouse monoclonal antibody for MSH2 (clone MX061), a rabbit monoclonal antibody for MSH6 (clone EP49), a mouse monoclonal antibody for MLH-1 (clone ES05), and a rabbit monoclonal antibody for PMS2 (clone EP51). Immunostaining was assessed independently by three experienced urological pathologists. MMR-related protein loss was defined by MMR-related protein loss in any tumor cells in any tumor spot, with intact staining in admixed benign prostate gland and/or surrounding stromal cells, endothelial cells, or lymphocytes. MMR-related protein staining without internal control staining was considered ambiguous and was not scored. The tumors were defined as hypermutated if the tumor mutation burden (TMB) > 10 mutations/Mb. Tumors were classified as showing MSI/dMMR if they were found to harbor a deleterious germline or somatic alteration in an MMR-related gene or had MMR-related protein loss by IHC.

### Microsatellite instability PCR

MSI-polymerase chain reaction (PCR) testing was performed by the genetics laboratory of the Department of Pathology of Ruijin Hospital, using the MSI analysis kit (AmoyDx, Xiamen, China) following the manufacturer instructions, for amplification of five mononucleotide repeat markers (CAT25, BAT-26, BAT-25, NR-24, MONO-27) and two pentanucleotide repeat loci (Penta-D and Penta-E), to confirm identity between the tumor and paired benign tissue. Status of MSI-high was given when ≥ two of these markers showed instability and status of MSI-low if only one marker showed instability, otherwise microsatellite stability (MSS) was assumed.

### Library preparation and targeted next-generation sequencing

Next-generation sequencing (NGS) was performed in a CLIA‐ and CAP‐accredited laboratory (Nanjing Geneseeq Technology Inc., Nanjing, China). Genomic DNA was extracted from formalin-fixed, paraffin-embedded (FFPE) samples using the QIAamp DNA FFPE Tissue Kit (Qiagen, Hilden, Germany), following the manufacturer’s instructions. Purified DNA was quantified by Qubit 3.0 using the dsDNA HS Assay Kit (Life Technologies, Carlsbad, CA, USA) and qualified by Nanodrop2000 (Thermo Fisher Scientific, Waltham, MA, USA). Sequencing libraries were prepared by KAPA Hyper Prep kit (KAPA Biosystems, Wilmington, MA, USA). A customized xGen lockdown-probe panel (Integrated DNA Technologies, Coralville, IA, USA) were used for selective enrichment for 437 predefined cancer-related genes (Geneseeq Technology, Inc., Toronto, ON, Canada; Prime panel). The capture reaction was performed with DynaBeads M-270 (Life Technologies) and xGen Lockdown Hybridization and Wash kit (Integrated DNA Technologies). The purified library was quantified by quantitative PCR using the KAPA Library Quantification kit (KAPA Biosystems), and its fragment size distribution was analyzed by Bioanalyzer 2100 (Agilent Technologies, Inc., Santa Clara, CA, USA). The target-enriched libraries were sequenced on a HiSeq4000 NGS platform (Illumina, San Diego, CA, USA) with 2 × 150-bp pair-end reads to primary coverage depths of 800×.

MSI was estimated based on 52 indel sites in the Geneseeq Prime panel. If > 40% of 52 sites showed unstable status (compared to the distribution in the 500 normal sample pools), the sample was defined as showing MSI. The TMB was defined as the number of somatic, coding, base substitution, and indel mutations per megabase of genome examined, and was calculated as previously described (20, 21).

## Results

### Prevalence and clinicopathological features of cases with MMR-related protein loss by immunohistochemistry

We screened for MMR-related protein loss in 1,141 primary PCas, including 88 GG1 tumors, 353 GG2 tumors, 190 GG3 tumors, 279 GG4 tumors, and 231 GG5 tumors. Altogether, 0.7% (8/1,141) of primary PCas had MMR-related protein loss (MSI-PCas), including 1.4% (4/279) GG4 and 1.5% (4/231) of GG5 tumors.

**Figure 1:**
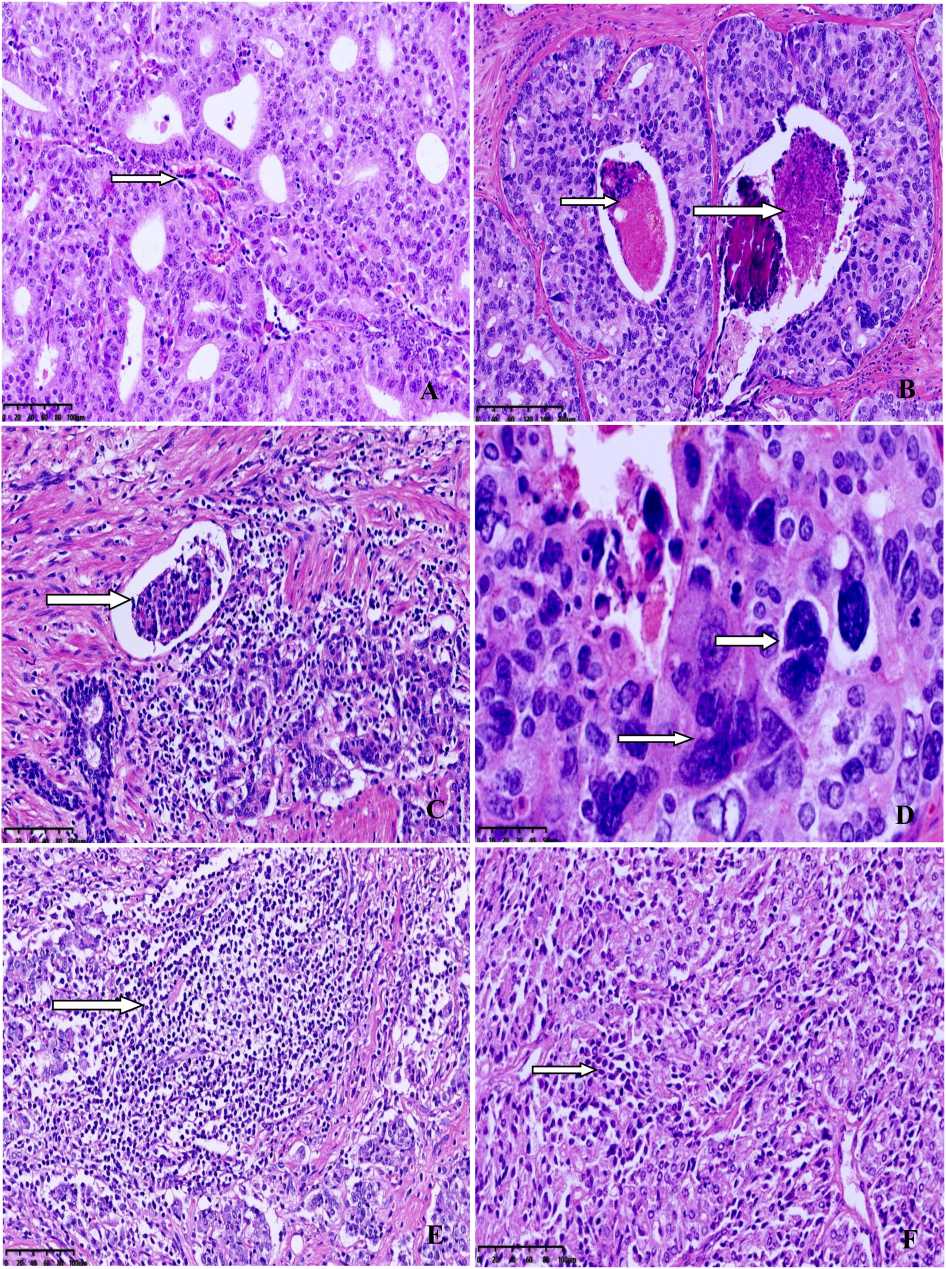
Representative morphology from selected cases. **(A)** Ductal adenocarcinoma with well-established fibrovascular cores. **(B)** Intraductal carcinoma with comedocecrosis. **(C)** Lymphovascular invasion. **(D)** Higher power view of pleomorphic giant-cells with bizarre atypia. **(E)** Dense lymphocytic infiltration in the tumor. **(F)** Diffuse lymphocytic infiltration between the tumor cells.

**Figure 2:**
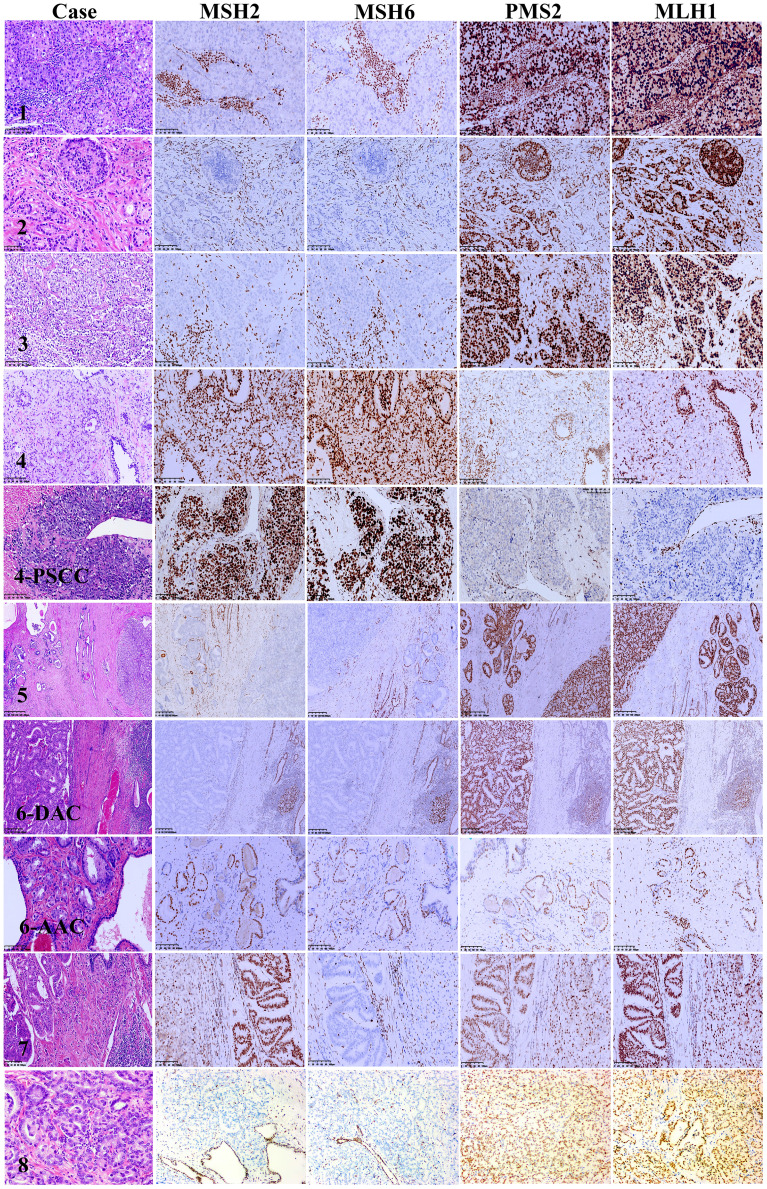
Mismatch repair protein immunohistochemistry in microsatellite instability prostate carcinoma. Case 1: MSH2 and MSH6 nuclear immunostaining are absent in tumor cells nuclei but present in infiltrating lymphocytes, PMS2 and MLH1 fully preserve expression. Case 2: MSH2 and MSH6 expression loss in both intraductal carcinoma and invasive carcinoma of the prostate, PMS2 and MLH1 overexpression in both intraductal carcinoma and invasive carcinoma of the prostate. Case 3: The high-grade invasive carcinoma of the prostate shows MSH2 and MSH6 loss with intact PMS2 and MLH1 nuclear expression. Case 4: Both the primary invasive carcinoma and small cell carcinomas after androgen deprivation therapy show intact MSH2 and MSH6 and lack of PMS2 and MLH1 nuclear staining. Case 5: Both intraductal carcinoma and invasive carcinoma of the prostate show MSH2 and MSH6 expression loss with PMS2 and MLH1 overexpression. Case 6: MSH2 and MSH6 expression loss in the ductal adenocarcinoma but not in the acinar adenocarcinoma. Both ductal adenocarcinoma and acinar adenocarcinoma show PMS2 and MLH1 overexpression. Case 7: Both the ductal adenocarcinoma and acinar adenocarcinoma show MSH6 loss with intact MSH2, PMS2 and MLH1 expression. Case 8: MSH2 and MSH6 expression loss, PMS2 and MLH1 overexpression. Lymphocytes, endothelial cells and stromal cells used as internal control.

The clinicopathological characteristics of the eight MSI-PCa cases are detailed in **Table 1**. Patients’ age at diagnosis ranged from 63 to 91 years (median: 72 years). The MSI-PCa tumors were highly aggressive based on pathological features, including tumor grade and stage. All cases had Gleason scores of 8 or 9. In addition to usual acinar adenocarcinoma (AAC), ductal/intraductal histology was present in 6 of the 8 patients (ductal in 4, **Figure 1A**, and intraductal in 2. **Figure 1B**). Of the six cases with pathological stage information at radical prostatectomy available, three presented with pT2c and three with pT3b (2 with nodal involvement). PNI was observed in all MSI-PCa patients. Two cases had LVI (**Figure 1C**). Four cases demonstrate focal pleomorphic giant-cell features (**Figure 1D**). All cases had a higher density of TILs within the tumor (**Figures 1E, F**). By IHC, six cases had loss of both MSH2 and MSH6, one (case 4) had loss of both MLH1 and PMS2, and one (case 7) had loss of MSH6 protein only (**Figure 2**, case1-8). Interestingly, the AAC area in case 6 showed intact MMR-related protein immunostaining (**Figure 2**, case 6). Six patients had received standard androgen-deprivation therapy (ADT), and four had received first-line abiraterone or enzalutamide. One patient received anti-PD-1/PD-L1 therapy. One patient (case 4) progressed to prostatic small cell carcinoma after 10-month ADT with TURP. The PSCC showed MLH1 and PMS2 protein loss, in keeping with the needle biopsy specimen (**Figure 2**, case 4). One patient developed bone metastases 4 months after radical prostatectomy (case 2).

**Table 1:** Clinicopathological characteristics of MSI prostate cancer.

Case	Age (years)	Specimen type	Treatment	pTNM	Gleason score (GG)	PNI	LVI	IDCP	DA, %	TILs	Pleo. cells	F/U (M)
1	91	TURP	ADT	NA	5 + 4 (5)	Present	Absent	Absent	10%	Present	Present	Death, 7
2	66	RP	ADT	pT2cN1	4 + 5 (5)	Present	Present	Present	Absent	Present	Absent	bone metastasis, 21
3	76	RP	ADT	pT3bN1	5 + 4 (5)	Present	Present	Absent	Absent	Present	Present	Survival, 27
4	67	Needle	ADT	NA	4 + 4 (4)	Present	Absent	Absent	20%	Present	Absent	Lung metastasis, 16
5	63	RP	ADT+anti-PD-1/PD-L1 therapy	pT2cNx	4 + 4 (4)	Present	Absent	Present	Absent	Present	Present	Survival, 21
6	68	RP	No adjuvant therapy	pT2cNx	DAC:4 + 4(4) AAC:3 + 3(1)	Present	Absent	Absent	60%	Present	Absent	Survival, 19
7	71	RP	ADT	pT3bN0	4 + 5 (5)	Present	Absent	Absent	50%	Present	Absent	Survival, 24
8	74	RP	NA	pT3bNx	4 + 4 (4)	Present	Absent	Absent	Absent	Present	Present	NA

AAC, acinar adenocarcinoma; ADT, androgen deprivation therapy; DAC, ductal adenocarcinoma; F/U, follow-up; GG, Grade Group; IDC-P, intraductal carcinoma of the prostate; LVI, lymphovascular invasion; M:month; NA, not available; Pleo., pleomorphic; PNI, perineural invasion; RP, radical prostatectomy; TILs, tumor infiltrating lymphocytes; TURP, transurethral resection of the prostate.

### Results of MSI-testing by PCR and Targeted NGS and correlation with IHC

**Figure 3:**
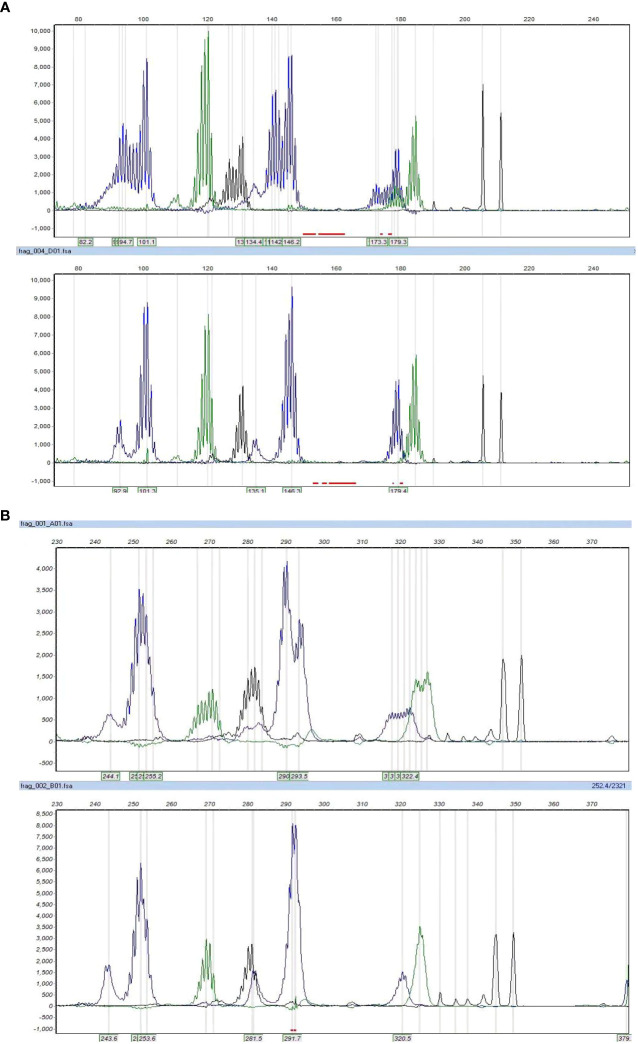
The microsatellite instability status by PCR. Cases 3 and 8 showed MSI-H, with allelic shifts in 4 of 7 markers in case 3 **(A)** and 7 of 7 markers in case 8 **(B)**.

**Figure 4:**
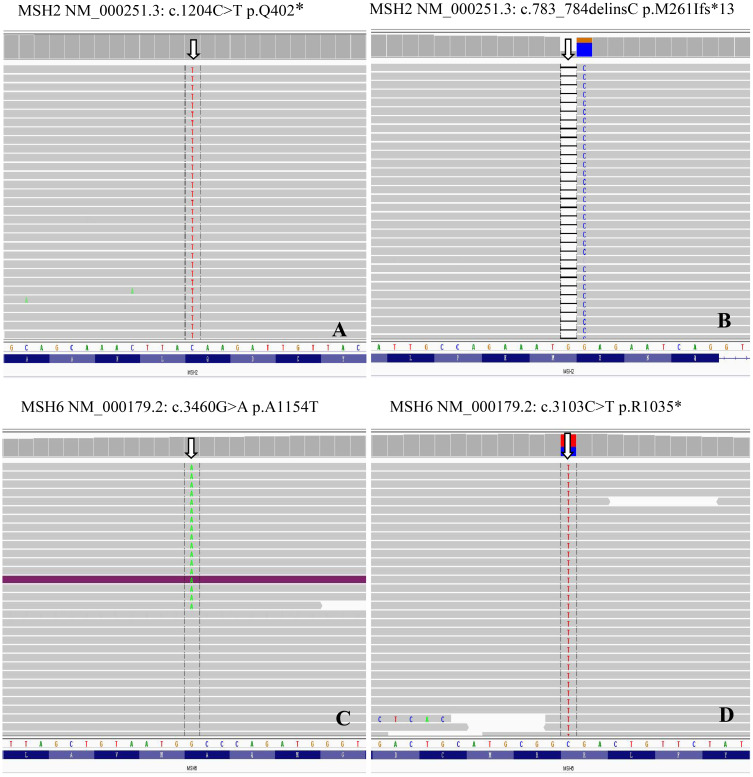
MMR genes mutations in MSI prostate cancer. Genomics Viewer screenshot of the representative MMR genes mutations, including MSH2 **(A)**, c.1204C>T p.Q402*; **(B)**, c.783_784delinsC p.M261Ifs*13) and MSH6 **(C)**, c.3460G>A p.A1154T; **(D)**, c.3103C>T p.R1035*).

**Table 2** summarizes the MSI status and MMR gene alterations. For the eight samples with MMR-related protein loss by IHC, the MSI status of seven cases were also evaluated by PCR, which was not performed for case 1, due to the lack of control paired benign tissue. Of the seven tumors tested for MSI by PCR, two were MSI-H (**Figures 3A, B**) and both had loss of MSH2 and MSH6 expression by IHC. By targeted NGS, 4 of the seven cases showed MSI and also had loss of MMR-related protein expression by IHC (3 with MSH2 and MSH6 protein loss and 1 with MLH1 and PMS2 protein loss). Of the 4 MSI-postive cases by targeted NGS, 3 had pathogenic somatic mutations in MMR-associated genes, including one with mutation in *MSH2* (case 3, **Figure 4A**), one with mutations in *MSH2* and *MSH6* (case 1, **Figures 4B, C**), and one with mutation in *MLH3* (case 4). Three cases (case 2, 6, and 7) demonstrated MMR-related protein loss by IHC but showed MSS by both PCR and targeted NGS. Of these 3 cases, case 6 showed a pathogenic germline mutation of *MSH6* (**Figure 4D**) and a pathogenic somatic mutation in *MSH2*, case 7 showed a pathogenic somatic mutation in *MSH6*, while case 2 demonstrated no mutations involving MMR genes.

**Table 2:** MSI status by IHC, PCR and targeted NGS, and MMR genes alterations.

Case	MMR proteins loss-IHC	MSI-PCR	MSI-NGS	MMR mutation	Coding Effect	Amino acid alteration	VAF	Somatic or germline status	Variant interpretation	Tumor mutation burden
1	MSH2/MSH6 loss	ND	MSI	MSH2	Frameshift	c.783_784delinsC p.M261Ifs*13	69.22%	Somatic	Likely pathogenic	71.1
				MSH6	Missense	c.3460G>A p.A1154T	3.11%	Somatic	Pathogenic	
2	MSH2/MSH6 loss	MSS	MSS	NA	NA	NA	NA	NA		58.7
3	MSH2/MSH6 loss	MSI-H	MSI	MSH2	Stop_gained	c.1204C>Tp.Q402*	16.05%	Somatic	Pathogenic	45.3
				POLD1	Missense	c.496C>T(p.R166W)	50.81%	Germline	VUS	
				POLD1	Missense	c.1621G>A(p.V541M)	11.93%	Somatic	VUS	
4	MLH1/PMS2 loss	MSS	MSI	MLH3	Frameshift	c.1755del(p.E586Nfs*24)	15.25%	Somatic	Likely pathogenic	39.1
				POLD1	Missense	c.2811G>T(p.M937I)	5.08%	Somatic	VUS	
5	MSH2/MSH6 loss	MSS	MSI	NA	NA	NA	NA	NA	NA	44.3
6 DAC	MSH2/MSH6 loss	MSS	MSS	MSH6	Stop_gained	c.3103C>T p.R1035*	53.92%	Germline	Pathogenic	25.7
				MSH2	Frameshift	c.229_230delp.S77Cfs*4	8.47%	Somatic	Pathogenic	
6 AAC	intact	MSS	MSS	MSH6	Stop_gained	c.3103C>T p.R1035*	44.22%	Germline	Pathogenic	0
7	MSH6 loss	MSS	MSS	MSH6	Frameshift	c.3305_3306insA p.G1105Wfs*3	39.01%	Somatic	Likely pathogenic	63.9
				MLH3	Synonymous	c.3960C>T(p.G1320=)	47.64%	Germline	Benign	
				POLD1	Missense	c.1418C>T(p.T473M)	2.96%	Somatic	VUS	
8	MSH2/MSH6 loss	MSI-H	ND	ND	ND	ND	ND	ND	ND	ND

AAC, acinar adenocarcinoma; DAC, ductal adenocarcinoma; MMR, mismatch repair; MSI, microsatellite instability; MSS, microsatellite stable; ND, not done; NA, not available; VUS, variant of unknown significance.

### Comprehensive analysis of somatic and germline alterations

**Tables 2**, **3** summarize the genomic characteristics. All cases with targeted NGS showed high TMB. The median TMB was 45.3 mutations/Mb (range, 25.7–71.1 mutations/Mb). Interestingly, the AAC component in case 6 had no tumor mutation burden. Somatic and germline alterations of relevant genes in MSI/dMMR-PCas are shown in **Figure 5**, and grouped in pathways that are potentially clinically actionable. Overall, MSI/dMMR-PCa cases showed a high frequency of alterations in genes in DNA damage repair (DDR) pathways and of germline mutations in DDR genes, including the homologous recombination repair (HRR), MMR, and other DDR pathways. Six patients (6/7) had a known or likely pathogenic somatic mutation in DDR pathway genes, including in *MSH2* (3/6), *MSH6* (3/6), *ATR* (2/6), *ATM* (2/6), *TP53* (2/6), *PTEN* (2/6), *MLH3* (1/6), *RAD50* (1/6), *PALB2* (1/6), *ERCC2* (1/6), *RAD54L* (1/6), and *ATRX* (1/6). Among seven patients carrying the pathogenic or likely pathogenic DDR gene mutations, five (71.4%) were involved in the MMR pathway, two (28.6%) were in the HRR pathway, and four (57.1%) were in other DDR pathways. Five patients (5/7) harbored a germline alteration in a gene that is involved in HRR pathway, including *BRCA2, RECQL4, PALB2, RAD54L, BRCA1, POLH*, and *RAD51D*. In total, 71.4% of patients (5/7) had pathogenic somatic or germline alterations in MMR genes, including *MSH2* (3/5), *MSH6* (3/5), *POLD1* (3/5), and *MLH3* (2/5). Two patients without evidence of MSI by PCR and targeted NGS had pathogenic or likely pathogenic germline and somatic MMR gene alterations One patient with MSI (case 5) had no pathogenic or likely pathogenic MMR genes and other DDR pathway gene alterations, however, the patient had likely pathogenic mutations in *ARID1A, JAK1*, and *APC*. The ductal adenocarcinoma (DAC) of case 6 also had a likely pathogenic mutation in *ARID1A* and pathogenic mutations in *BRAF* and *JAK3*.

**Table 3:** Somatic and germline alterations in DNA damage repair genes and other genes mutations.

Case	Other DDR genes mutations	Coding Effect	Amino acid alteration	VAF	Somatic or germline status	Variant interpretation	Other mutations of interest
1	MRE11	Missense	c.1715G>A p.R572Q	46.81%	Germline	VUS	SPOP(c.391T>G p.W131G)
	ATR	Missense	c.3572T>G p.L1191W	44.89%	Germline	VUS	APC(c.4666dup p.T1556Nfs*3)
	ERCC5	Missense	c.3553A>G p.K1185E	52.22%	Germline	VUS	FOXA1(c.798_799insCAG p.F266_K267insQ)
	TP53	Trucation	c.1024C>T p.R342*	73.58%	Somatic	Pathogenic	JAK1(c.1289dup p.L431Vfs*22)
	PTEN	Missense	c.517C>T p.R173C	63.27%	Somatic	Pathogenic	JAK1(c.2580del p.K860Nfs*16)
	ATRX	Deletion	c.6792_6794del p.E2265del	19.25%	Somatic	VUS	TSC1(c.3065G>A p.R1022K)
	ATR	Frameshift	c.6618dup p.S2207Ifs*15	7.84%	Somatic	Likely pathogenic	ERBB3(c.395G>A p.R132H)
2	FANCI	Missense	c.2875C>T p.R959W	48.23%	Germline	CIP:VUS; Likely benign	FOXA1(c.781C>G p.R261G)
	RAD50	Trucation	c.3553C>T p.R1185*	29.93%	Somatic	Pathogenic	TSC2(c.5138G>A p.R1713H)
	PALB2	Frameshift	c.3026del p.P1009Lfs*6	25.20%	Somatic	Pathogenic	SETD2(c.1270C>T p.R424*)
	ATR	Frameshift	c.6772dup p.I2258Nfs*22	23.76%	Somatic	Likely pathogenic	KDM5A(c.3597del p.G1200Dfs*9)
	CUL3	Missense	c.113C>T p.T38M	23.24%	Somatic	VUS	MTOR(c.3428C>T p.T1143M)
	ATR	Missense	c.4892G>A p.R1631H	22.54%	Somatic	VUS	RNF43(c.1976del p.G659Vfs*41)
	WRN	Splicing	c.356-3_356-2del	18.08%	Somatic	VUS	ERBB3(c.1561C>T p.R521*)
	BRCA2	Missense	c.8662C>T p.R2888C	18.07%	Somatic	Benign​	MTOR(c.4831C>T p.R1611*)
	ATM	Missense	c.8667T>A p.D2889E	9.82%	Somatic	Likely pathogenic	KMT2A(c.1279C>T p.R427W)
	XRCC2	Trucation	c.190C>T p.R64*	2.27%	Somatic	Pathogenic	TSC1(c.1217A>G p.Y406C)
3	POLD1	Missense	c.496C>T p.R166W	50.81%	Germline	VUS	JAK3(c.2771G>A p.S924N)
	PALB2	Missense	c.1213C>G p.P405A	50.00%	Germline	VUS	PBRM1(c.2627G>A p.R876H)
	ERCC1	Missense	c.871G>A p.E291K	12.20%	Somatic	VUS	FOXA1(c.355C>T p.Q119*)
	POLD1	Missense	c.1621G>A p.V541M	11.93%	Somatic	VUS	JAK1(c.2580del p.K860Nfs*16)
	BRCA2	Missense	c.521G>A p.R174H	11.69%	Somatic	CIP:VUS; Likely benign	JAK1(c.1289del p.P430Rfs*2)
	RAD54L	Missense	c.1625G>A p.R542H	10.38%	Somatic	Likely pathogenic	SPOP(c.397T>G p.F133V)
	PARP1	Missense	c.413G>A(p.R138H)	10.21%	Somatic	VUS	
	CHEK2	Missense	c.725T>C p.F242S	10.22%	Somatic	VUS	
	SMARCA4	inframe_deletion	c.708_713del(p.G243_P244del)	9.69%	Somatic	VUS	
4	BRCA1	Missense	c.824G>A p.G275D	55.37%	Germline	Benign	FOXA1(c.837_842dup p.S282_G283dup)
	FANCI	Missense	c.236G>A p.G79E	51.02%	Germline	VUS	KDM5A(c.3597del p.G1200Dfs*9)
	RECQL4	Missense	c.1561C>T(p.R521W)	33.56%	Germline	VUS	EZH2(c.1774_1777del p.T592Vfs*82)
	ATRX	Frameshift	c.76_88del p.S26Kfs*11	48.65%	Somatic	Likely Pathogenic	TSC2(c.170G>A p.R57H)
	TP53	Missense	c.799C>T(p.R267W)	38.33%	Somatic	Pathogenic	TSC1(c.3127_3129del p.S1043del)
	PTEN	Frameshift	c.800del(p.K267Rfs*9)	28.69%	Somatic	Pathogenic	TMPRSS2::ERG fusion
	FANCI	Missense	c.1637A>C p.N546T	21.74%	Somatic	VUS	
	FANCL	Missense	c.877C>T p.P293S	6.59%	Somatic	VUS	
	POLD1	Missense	c.2811G>T p.M937I	5.08%	Somatic	VUS	
5	RAD54L	Missense	c.788G>A p.G263E	44.57%	Germline	VUS	ARID1A(c.3216del p.K1072Nfs*21)
							PBRM1(c.2786A>G p.E929G))
							JAK3(c.1820C>T(p.A607V))
							JAK1(c.2264G>A(p.R755Q))
							FOXA1(c.677A>T(p.D226V)
							JAK1(c.1289dup(p.L431Vfs*22)
							JAK1(c.1289del(p.P430Rfs*2)
							JAK1(c.2580del(p.K860Nfs*16)
							RET(c.1595G>T(p.G532V);RET(c.1183G>A(p.V395M)
	FANCA	intron_variant	c.2778+10C>T	42.92%	Germline	CIP:VUS; Likely benign	APC(c.4643del(p.N1548Tfs*17)
	POLE	Missense	c.5347G>C p.D1783H	42.61%	Germline	VUS	APC(c.4384_4385del(p.K1462Efs*6)
	POLE	Missense	c.5312C>T p.T1771M	6.86%	Somatic	VUS	KDM5A(c.4074+2T>C);KDM5A(c.3052G>A p.A1018T))
6 DAC	MUTYH	Missense	c.976G>A(p.V326M)	50.51%	Germline	VUS	ARID1A(c.1650dup p.Y551Lfs*72)
	RAD51D	Missense	c.196G>A p.V66M	47.92%	Germline	CIP:VUS; Benign; Likely benign	BRAF(c.1801A>G p.K601E)
	BRCA2	Missense	c.8474C>A p.A2825E	37.84%	Germline	VUS	FOXA1(c.752_763del(p.G251_F254del)
	POLE	Missense	c.6119C>T p.A2040V	11.31%	Somatic	VUS	JAK3(c.1204C>T(p.R402C)
6 ACC	RAD51D	Missense	c.196G>A p.V66M	42.63%	Germline	CIP:VUS; Benign; Likely benign	NA
	MUTYH	Missense	c.976G>A(p.V326M)	37.76%	Germline	VUS	
	BRCA2	Missense	c.8474C>A p.A2825E	34.93%	Germline	VUS	
					
7	POLH	Missense	c.1694A>G p.N565S	50.92%	Germline	VUS	FOXA1(c.784C>T(p.R262C)
	RECQL4	Missense	c.3509C>T(p.P1170L)	49.62%	Germline	VUS	BRAF(c.1801A>G(p.K601E)
	NTHL1	Missense	c.556G>A(p.A186T)	47.77%	Germline	VUS	JAK2(c.2936C>T(p.T979M)
	MLH3	Synonymous	c.3960C>T p.G1320=	47.64%	Germline	VUS; Benign	KMT2B(c.7777C>T(p.R2593C)
	ERCC5	Missense	c.3145G>C p.D1049H	46.52%	Germline	VUS	JAK1(c.1825G>A(p.E609K)
	POLE	Missense	c.4168C>T p.R1390C	27.49%	Somatic	VUS	TSC2(c.3380G>A(p.R1127Q)
	ATM	Missense	c.8122G>A p.D2708N	20.10%	Somatic	Likely pathogenic​	FOXA1(c.1392T>A(p.Y464*)
	POLD1	Missense	c.1418C>T p.T473M	2.96%	Somatic	VUS	TSC2(c.5207A>G(p.Y1736C)
8	ND		ND	ND	ND	ND	ND

AAC, acinar adenocarcinoma; CIP, conflicting interpretations of pathogenicity; DAC, ductal adenocarcinoma; DDR, DNA damage repair; NA, not available; ND, not done; VUS, variant of unknown significance.

**Figure 5:**
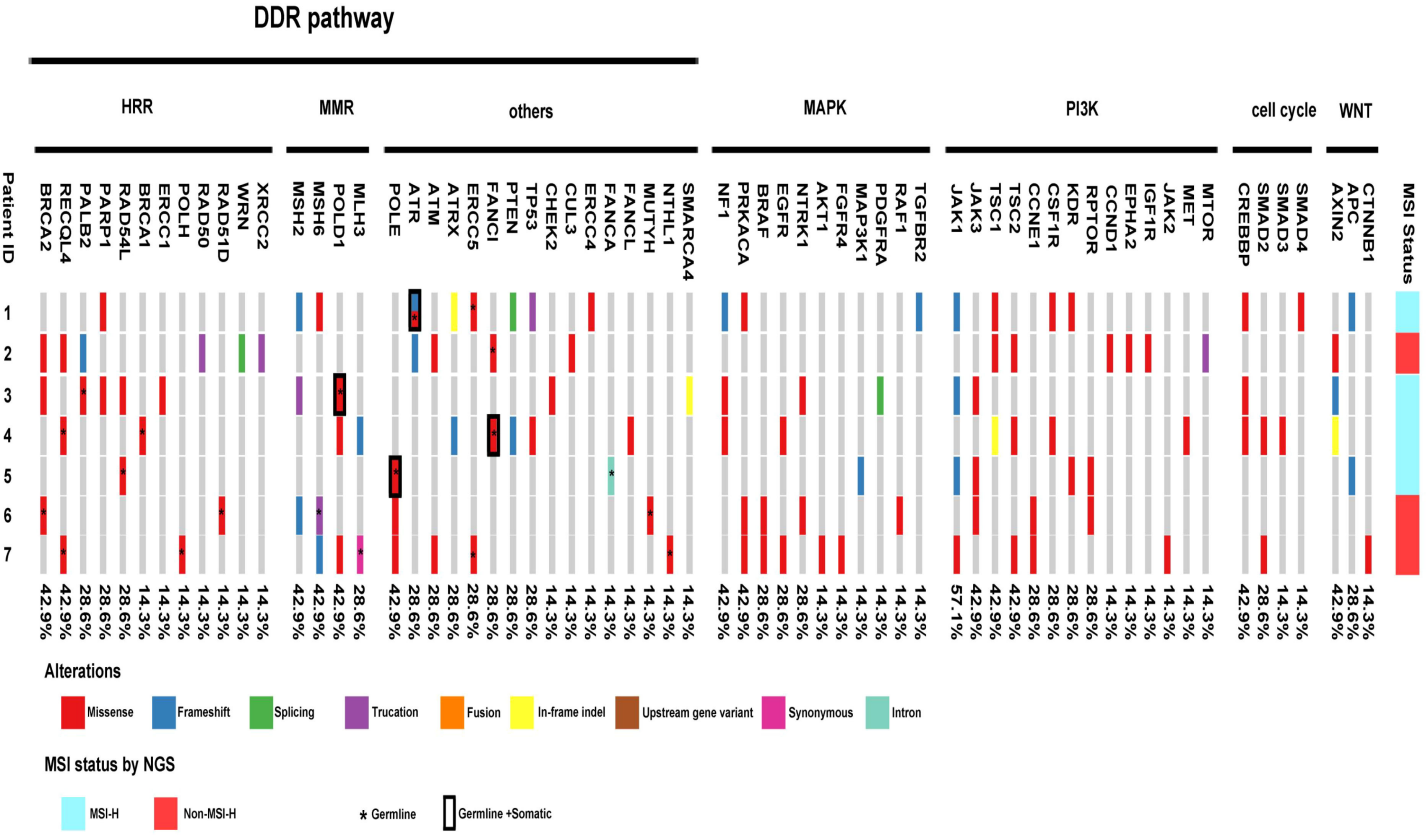
Landscape of genomic alterations across MSI prostate cancer, including alterations in genes involved in DDR pathway, MAPK pathway, PI3K pathway, Cell cycle and Wnt-βcatenin pathway. Among seven patients carrying the pathogenic or likely pathogenic DDR gene mutations, five (71.4%) were involved in the MMR pathway, two (28.6%) were in the HRR pathway, and four (57.1%) were in other DDR pathways. 85.7% of patients (6/7) harbored somatic alterations in MAPK pathway genes. All patients carried somatic alterations in the PI3K pathway. Four of the seven patients harbored somatic alterations in cell-cycle pathway genes. Six of the seven patients carried somatic alterations in the WNT/β-catenin pathway.

In addition, 85.7% of patients (6/7) harbored somatic alterations in mitogen-activated protein kinase (MAPK) pathway genes, including mutations in *NF1* (3/6), *PRKACA* (3/6), *BRAF*(2/6), *EGFR* (2/6)*, NTRK1* (2/6), *AKT1* (1/6), *FGFR4* (1/6), *MAP3K1* (1/6), *PDGFRA* (1/6), *RAF1* (1/6), and *TGFBR2* (1/6). All patients carried somatic alterations in the PI3K pathway, including hot spot mutations in *JAK1, JAK3, TSC1*, and *TSC2*. Four of the seven patients harbored somatic alterations in cell-cycle pathway genes, including *CREBBP, SMAD2, SMAD3*, and *SMAD4*. Six of the seven patients carried somatic alterations in the WNT/β-catenin pathway, including *AXIN2, APC*, and *CTNNB1*. Notably, one patient (1/7, case 4) had *TMPRSS2::ERG* fusion.

## Discussion

In the current study, we reported a 0.7% prevalence rate of MSI/dMMR tumors among primary PCa patients in the Chinese population. The identified MSI/dMMR-PCas showed a high frequency of DDR pathway gene mutations, including five cases with pathogenic somatic or germline MMR gene mutations. Activating mutations in the MAPK pathway, PI3K pathway, and WNT/β-catenin pathway were common, while a *TMPRSS2::ERG* rearrangement was identified in one case (case 4).

MSI/dMMR has been detected in 3% of unselected PCa cases (9, 22) and in 5% of metastatic PCa cases (10, 14). In a study of 60 advanced PCas, 12% (7/60) had MMR gene mutations and MSI (16). The MSI-H/dMMR frequency was found to be 3.1% in a large series of study that included 1,033 PCa patients and used NGS (9). Guedes et al. (15) reported MSH2 protein loss in primary PCas, whereas MLH1, PMS2, and MSH6 were not evaluated. In their study, 1.2% (14/1,176) of PCas had MSH2 loss, including 1% (12/1133) of primary adenocarcinomas and 5% (2/43) of PSCCs (15). In a recent study by Fraune et al. (22), 3.5% (7/200) of advanced PCas were MSI/dMMR based on IHC. Recently, Kagawa et al. (23) investigated the prevalence of dMMR-PCas in the Japanese population. They found that 1.2% (4/337) showed loss of MMR-related protein expression by IHC. The overall prevalence of MSI/dMMR-PCas in the Chinese population is thus extremely low, probably due to differences in the cohorts selected (our study has included all Gleason score groups) or racial differences.

The role of MMR-related proteins has been extensively studied in colorectal and endometrial cancer. Few reports have analyzed dMMR-PCa by IHC (15, 22, 24–28). We here analyzed the immunohistochemical expression of MMR-related proteins in a large series of PCas. To our knowledge, no previous study has examined the phenotype of sporadic primary PCas with dMMR in a large number of Chinese specimens. Among the MSI/dMMR patients in our study (8/1,141), six of eight patients demonstrated combined loss of MSH2 and MSH6, which was consistent with the studies by Kagawa et al. (23) (4/4) and Fraune et al. (22) (6/7).The combined loss pattern of MSH2 and MSH6 may be more frequent in PCas, while it is rather infrequent in colorectal and endometrial cancer (29). All of the dMMR-PCa cases in our series were high-grade PCas, including 1.4% (4/279) of tumors with GG4 and 1.5% (4/231) of tumors with GG5. The high incidence of dMMR in higher grade PCas in the present study also agrees with previous findings. As reported by Guedes et al. (15), 8% (7/91) of tumors with primary Gleason pattern 5 (5 + 4 = 9 or 5 + 5 = 10) PCas had MSH2 loss, while <1% of tumors from lower Gleason score groups had MSH2 loss. Kagawa et al. (23) also reported patients with dMMR PCas were at a significantly higher stage and had a greater Gleason score (≥8) than those with proficient MMR PCas. Recently, Wyvekens et al. (30) evaluated the histopathological features of 19 dMMR PCas and found that all treatment-naive dMMR cases (11/11, 100%) were GG 4 or 5. These findings suggest that patients with high-grade primary PCas (Gleason score ≥ 8) are more likely to harbor dMMR/MSI, which argues the need for routine clinical screening for MMR gene loss using IHC.

The morphological and molecular correlates of dMMR have been recognized in several tumor types, such as colorectal adenocarcinoma, endometrial adenocarcinoma, and upper tract urothelial carcinoma (11, 31, 32), whereas the histopathological features of dMMR primary PCas remain unclear. Recently, several morphological features have been reported as associated with dMMR-PCa, such as ductal/intraductal carcinoma, pleomorphic giant-cell features, LVI, cribriform and/or solid growth patterns, and TILs (15, 18, 19, 30, 33). Ductal/intraductal carcinoma (60%) and pleomorphic giant-cell features (50%) were frequently present in dMMR PCas. Prominent TILs were identified in all our dMMR-PCa cases. Guedes et al. (15) found that tumors with MSH2-loss had a higher density of infiltrating CD8^+^-lymphocytes than did grade-matched controls without MSH2-loss. Recently, Wyvekens et al. (30) also reported that TILs was a histopathological feature of dMMR-PCas (7/11, 64%).As we know, prominent intra- and peritumoral lymphocytic infiltrates are common in dMMR colorectal carcinomas (31). TILs might be a useful histopathological feature for identifying selected PCas for MMR-related protein IHC and genetic testing. Recently, TMB has also been proposed as a biomarker of response to immunotherapy (34). All of dMMR PCa patients in our study showed high TMB (median: 45.3 mutations/Mb, range: 25.7–71.1 mutations/Mb). Further studies examining the role of varied histological features, TIL density, or TMB in predicting sensitivity to immunotherapy should be considered.

We confirmed that dMMR-PCa cases are enriched for actionable mutations and found a significantly higher frequency of changes in DDR pathway-related genes, including mutations in MMR-related genes. Targeted NGS revealed pathogenic or pathogenic somatic mutations in MMR genes, including *MSH2* (3/5) and *MSH6* (2/5) in five of 7 dMMR-PCa cases. One dMMR PCa patient was identified as having a pathogenic germline *MSH6* mutation. Pritchard et al. (16) demonstrated that MSI-advanced PCas are frequently driven by complex structural *MSH2* or *MSH6* rearrangements, rather than by MLH1 epigenetic silencing. None of the MMR mutations in our study were inherited in the germline. In a larger study of 1,133 primary PCas, a small percentage (1.2%) demonstrated MSH2 loss by IHC, with confirmation by NGS, while only three patients had germline mutations (15). Recently, Abida et al. (9) revealed genetic alterations in MSH2 and in MSH6 in 46% each of MSI-H/dMMR PCas, whereas the mutations rates for *PMS2* and *MLH1* were significantly lower (< 20% each), and seven of the 32 MSI-H/dMMR patients (21.9%) had a pathogenic or likely pathogenic germline mutation in MMR genes, including five in *MSH2*, one in *MSH6*, and one in *PMS2*. Furthermore, Wyvekens et al. (30) evaluated the molecular features of 19 dMMR-PCas and demonstrated that dMMR was secondary to functional loss of MSH2/MSH6 and MLH1/PMS2 in 15 (79%) and four cases (21%), respectively, while germline mutations were present in four cases (4 of 19, 21%). Our study further suggests that somatic MMR mutations are more common than germline mutations in PCas, and that inactivation of MSH2 and MSH6 appears to be the main cause of MSI in PCas, in contrast to colorectal and endometrial cancer, where MSI is most often due to epigenetic silencing of *MLH1* (11, 12).

Most interestingly, in addition to MMR gene alterations, other pathogenic or likely pathogenic somatic mutations in DDR pathway genes in our dMMR-PCa cases included mutations in *TP53, PTEN, ATR, RAD50, PALB2, ATM, XRCC2, RAD54L*, and *ATRX*. Furthermore, some genes were recurrently mutated, including those involved in the MAPK and PI3K signaling pathways. These findings were consistent with a recent report examining genomic characterization of DAC: three of seven patients with MMR alterations also had concurrent secondary mutations in HRR pathway-related genes (35). Approximately 50% of primary PCas harbor *ETS* rearrangements, most commonly *TMPRSS2::ERG*. Interestingly, only one case in our MSI-PCas group showed *TMPRSS2::ERG* rearrangement (35). In a recent study of 19 dMMR-PCa cases, *TMPRSS2::ERG* fusions were detected in only two cases (2/19, 11%) (30). These results suggested that *TMPRSS2::ERG* fusions are infrequent in MSI/dMMR-PCas. *TMPRSS2::ERG* fusion is dependent on androgen receptor (AR) signaling and is thought to be restricted to the prostate (36). These data may suggest that AR-directed therapy is not absolutely required for MSI/dMMR-PCa patients.

Recent studies have shown a meaningful relationship of ductal histology with DDR genes alterations as well as MSI/dMMR status (18, 19, 35, 37). A relatively large sample size-based study by Schweizer et al. (35) showed that approximately 50% (25/51) of DAC patients demonstrated DDR pathway alterations. In our case 6, the DAC component accounted for about 60% of the tumor, and the DAC and AAC components were clearly separated. We separately sequenced the AAC and DAC component and the results showed that only the DAC component had somatic pathogenic mutations in MMR genes, with a higher frequency of gene alterations. This case suggested that concurrent DAC and AAC may share different gene alterations, as well as different changes in DDR genes between these components.

The MSI/dMMR status may be discordant when different assessment methods are used. In our study, eight PCas demonstrated loss of MMR-related protein expression by IHC. However, only two of the seven PCas showed MSI-H by multiplex PCR-based testing. MSI-PCR typically targets five informative microsatellite markers in the standard molecular diagnosis of MSI, although MSI-PCR has been validated for colorectal tumors and may have poor performance in other cancer types (38). In a previous study, MSI-PCR achieved only 81.8% sensitivity for PCas and 75.0% sensitivity for endometrial tumors (39). Similar observations have been made by Guedes et al. (15) and Fraune et al. (22), who found that only 61% (8/13) and 66.7% (4/6) of advanced dMMR-PCas exhibited MSI by MSI-PCR. Moreover, TILs may lead to false-negative MSI-PCR results. Two of the five discrepant PCas in our cohort evidenced MSI by NGS-based MSI testing, indicating false negative MSI-PCR results. In addition, two prostate tumors carried somatic pathogenic or likely pathogenic mutations in MMR genes by NGS, similarly indicating MSI-PCR was inaccurate. Interestingly, one PCa showed MMR-related protein loss, but no MSI by MSI-PCR or NGS. The optimal method for determining MSI/dMMR status in PCa patients remains unknown, however, our study suggests that NGS may represent a robust and efficient strategy to identify the subset of PCa patients.

Data regarding the response of dMMR PCa to standard treatment are conflicting. Some studies reported response and survival outcomes to standard therapies, which were similar to those reported in unselected patients, while other studies showed poor response to hormonal treatments in dMMR PCa patients (40). Considering the low number of patients included in these studies and the different methods used to assess MMR/MSI status, no firm conclusion can be drawn regarding the response to standard treatments of dMMR PCa. Immunotherapy-based approaches have enriched the therapeutical opportunities of many cancer types, improving patient survival; and in advanced PCa patients, there were several retrospective small series data on the treatment response of dMMR/MSI-H PCas to immunotherapy. Abida et al. (9) reported a PSA50 response rate of 54.5% in 11 patients with dMMR/MSI-H PCa with a durable response. A PSA50 response of 50% was also found by Antonarakis et al. (10) in 4 patients with MMR-mutated advanced PCa treated with anti-PD1 immunotherapy. Graham et al. (41) documented a similar PSA50 response rate of 53%. In addition, Nava Rodrigues et al. (42) have demonstrated a higher frequency of immunopositivity to antibody against to PD-L1 carboxy-terminal domain in dMMR PCas (50%) than in pMMR PCas (9.8%). Overall, these studies suggest that immunotherapy has promising prospects for treating advanced, dMMR/MSI-H PCas.

Our study had several limitations. First, it was a retrospective study exclusively from ethnic Chinese sample, and the number of patients with MSI was limited. Furthermore, it was not designed to assess the diagnostic accuracy of MSI in MMR-related proteins by IHC, which is prone to technical artifacts and may cause false-negative results when point mutations, missense mutations, or some protein-truncating mutations affect MMR genes. Finally, information on responses to anti-PD-1/PD-L1 immunotherapy was unavailable.

In conclusion, our study demonstrated that the MSI/dMMR phenotype is uncommon in PCas and that several clinicopathological features may be associated with the presence of MSI/dMMR or DDR mutations, in particular TILs. We propose that genetic testing should be considered for all PCas with TILs. NGS assays perform better than MSI-PCR for detecting MSI in PCas. Although MSI/dMMR-PCas are uncommon, they are clinically important, as this study strongly suggests that MSI/dMMR-PCas are enriched for actionable mutations. Cases with PCas with some clinicopathological features (such as intraductal/ductal histology and TILs) should be offered NGS to guide treatment.

## Data availability statement

The datasets presented in this study can be found in online repositories. The name of the repository and accession number can be found below: NCBI Sequence Read Archive; PRJNA1010485.

## Author contributions

HZ: Writing – original draft. XY: Writing – original draft. JX: Writing – original draft. XC: Writing – review & editing. JC: Conceptualization, Writing – review & editing. MS: Conceptualization, Writing – original draft. WD: Conceptualization, Writing – review & editing. SW: Conceptualization, Writing – review & editing. ZZ: Conceptualization, Writing – review & editing. CW: Writing – review & editing. MZ: Conceptualization, Validation, Writing – review & editing, Writing – original draft.
